# Role of aspartate aminotransferase in predicting Pediatric carbon monoxide poisoning outcomes: a Multicenter retrospective cohort study

**DOI:** 10.1093/toxres/tfaf154

**Published:** 2025-11-12

**Authors:** Xin-Hong Lin, Hsiu-Yung Pan, Wei-Ting Wu, Chih-Min Tsai, Ye-In Chang, Po-Chun Chuang

**Affiliations:** Department of Emergency Medicine, Kaohsiung Chang Gung Memorial Hospital, No. 123, Dapi Road, Niaosong District, Kaohsiung City 833, Taiwan (R.O.C.); Department of Emergency Medicine, Kaohsiung Chang Gung Memorial Hospital, No. 123, Dapi Road, Niaosong District, Kaohsiung City 833, Taiwan (R.O.C.); Department of Emergency Medicine, Kaohsiung Chang Gung Memorial Hospital, No. 123, Dapi Road, Niaosong District, Kaohsiung City 833, Taiwan (R.O.C.); Department of Computer Science and Engineering, National Sun Yat-sen University, No. 70, Lien-hai Road, Gushan District, Kaohsiung City 80424, Taiwan (R.O.C.); Department of Pediatrics, Kaohsiung Chang Gung Memorial Hospital, No. 123, Dapi Road, Niaosong District, Kaohsiung City 833, Taiwan (R.O.C.); College of Medicine, Chang Gung University, No. 259, Wenhua 1st Road, Guishan District, Taoyuan City 33302, Taiwan (R.O.C.); Department of Computer Science and Engineering, National Sun Yat-sen University, No. 70, Lien-hai Road, Gushan District, Kaohsiung City 80424, Taiwan (R.O.C.); Department of Emergency Medicine, Kaohsiung Chang Gung Memorial Hospital, No. 123, Dapi Road, Niaosong District, Kaohsiung City 833, Taiwan (R.O.C.); Department of Computer Science and Engineering, National Sun Yat-sen University, No. 70, Lien-hai Road, Gushan District, Kaohsiung City 80424, Taiwan (R.O.C.)

**Keywords:** carbon monoxide poisoning, aspartate aminotransferase, Pediatric

## Abstract

This multicenter retrospective cohort study investigated prognostic markers for clinical outcomes and delayed neurological sequelae (DNS) in pediatric patients with carbon monoxide poisoning (COP). We analyzed data from patients under 17 yr of age who presented to emergency departments between January 2007 and October 2018 with a carboxyhemoglobin (COHb) level exceeding 10%. A total of 162 cases were included. Clinical and laboratory parameters such as oxygen saturation (SpO₂), Glasgow Coma Scale (GCS), white blood cell (WBC) count, aspartate aminotransferase (AST), red cell distribution width (RDW), and COHb levels were evaluated. Poor outcomes were defined as death or a GCS score below 13 at discharge. Patients with poor outcomes had significantly lower SpO₂ and GCS scores, and elevated WBC, AST, and RDW levels (all *P* < 0.001). Among these biomarkers, AST had the highest area under the receiver operating characteristic curve (AUC = 0.879), and an AST cut-off value of 38.5 U/L yielded a sensitivity of 81.25% (95% CI: 54.35%–95.95%) and specificity of 87.10% (95% CI: 79.89%–92.44%) for predicting poor outcomes. Of the 148 patients with follow-up data, 10 developed DNS. These patients had significantly higher COHb levels (*P* = 0.032) and lower GCS scores at discharge (p = 0.002). Our findings suggest that AST is a strong and accessible biomarker for predicting poor outcomes in pediatric COP and may assist clinicians in early risk stratification and management.

## Introduction

Carbon monoxide poisoning (COP) is a common cause of severe poisoning in children worldwide.^1^ Common causes of COP are fire accidents, incomplete combustion in water heaters, or use of combustion equipment in poorly ventilated areas.^2^ In the United States, approximately 40,000 people require medical attention for COP annually, and approximately 5,000–6,000 fatalities.^2^ An increasing trend in COP has also been observed in Taiwan.^3^^,^^4^ Previous studies have shown that up to 40% of patients experience sequelae, primarily late neurocognitive impairment, following COP.^5^ The mortality rate associated with COP can be as high as 30%.^6^

CO has a higher affinity for hemoglobin (Hb) than for oxygen and forms carboxyhemoglobin (COHb) after binding to Hb, which disrupts the transport of oxygen, resulting in tissue hypoxia and subsequent oxidative stress and inflammation.^7^ Since the neurological and cardiovascular systems are sensitive to hypoxia, the clinical symptoms of COP in the acute phase often present as headache, dizziness, nausea, general weakness, chest tightness, and breathing difficulty. Severe COP may result in confusion, shock, or even death.^7^^,^^8^ Approximately 10%–30% of patients with acute COP may develop delayed neurological sequelae (DNS) within 3–240 days, leading to persistent deficits in executive functions.^5^ The exact mechanism of DNS is not yet well understood but is believed to be related to hypoxia and a profound demyelinating process affecting the lipid-containing myelin sheaths and cellular membranes.^9^^,^^10^ Pediatric patients have a higher metabolic rate and increased oxygen consumption relative to body weight, making them more susceptible to hypoxia-induced neurological sequelae. An immature central nervous system makes children vulnerable to COP, and DNS can result in poor performance, chronic headaches, memory impairments, and other neuropsychological symptoms in pediatric patients.^11^ Additionally, pediatric patients may not effectively communicate their symptoms, leading to delays in diagnosis and treatment.

Administration of 100% oxygen is used to restore the oxygen-carrying capacity of Hb by displacing CO from Hb and accelerating COHb metabolism in CO-intoxicated patients.^2^^,^^12^ The half-life of COHb in the body is approximately 320 min in ambient air^5^ and can be reduced to approximately 90 min with 100% oxygen and 20 min with a hyperbaric oxygen supply.^5^^,^^13^ Hyperbaric oxygen therapy (HBOT) has been reported to lower the risk of cognitive sequelae and reduce both short-term and long-term mortality rates in patients with severe COP who are under 20 yr of age or who present with acute respiratory failure.^7^^,^^13^^,^^14^

Previous studies have shown that initial loss of consciousness, acute respiratory failure requiring intubation, and the time from poisoning to the initiation of HBOT are risk factors for developing DNS and early death in COP patients.^8^^,^^15–17^ Laboratory examinations such as, white blood cell (WBC) count; COHb, neutrophil, lymphocyte, lactate, creatine kinase, N-terminal pro B-type natriuretic peptide (NT-proBNP), and troponin-I levels; and red cell distribution width (RDW), were used to predict the severity and prognosis of COP in previous studies^18–22^; While their predictive value remains inconsistent. Therefore, this study aimed to explore more reliable predictors of poor outcomes and delayed neurological sequelae in children with COP, focusing on the potential role of AST.

## Method

This retrospective study was approved by the Foundation Institutional Review Board (202400597B0).

### Study setting

This study was conducted by searching for key laboratory examinations (“COHb, AST, RDW, WBC count, lactate and troponin levels”) in the electronic medical records of the XXX system. It contained data from four XXX branches located across Taiwan: Keelung, Linkou, Chiayi, and Kaohsiung. A total of 162 patients were enrolled after a thorough review of their medical records by the first author. For the sub-analysis of DNS, 14 patients (11 deceased, 3 lost to follow-up) were excluded, resulting in 148 patients eligible for outpatient follow-up assessment.

### Patients

Patients under 17 yr of age who were brought to the emergency department (ED) between January 2009 and October 2018 because of CO exposure with COHb levels, which analyzed by Siemens RapidLab 1,265 Electrolyte and Blood Gas Analyzer greater than 10% were enrolled in the study. All patients received HBOT during the acute phase (within 1–2 wk) at 2.0 ATA—180 min if critically ill, or 90 min if stabilized. If DNS developed within 1 mo, further HBOT was provided based on clinical and neurological assessments, with treatment evaluated in 10-session units, up to a maximum of 40 sessions.

### Measurements

The time from CO exposure to ED visit was recorded. If the exact time was clearly documented in medical records, it was used directly; otherwise, we defined the approximate times as follows: “just now” was viewed as exposure within 1 h; “for hours” was viewed as exposure within 1–6 h; “yesterday,” “last night,” or “for 1 day” was regarded as exposure in 6–24 h; and “for days” was regarded as more than 24 h following exposure if the time period was not definitely described in the medical records. The times were categorized into four groups: less than 1, 1–6, 6–24, and > 24 h. The mechanism of the COP was grouped into incomplete fuel combustion, fire scenes, defective heaters, and others. Inevitable death upon discharge was defined as mortality. A poor outcome was defined as a Glasgow Coma Scale (GCS) score < 13 at discharge or mortality (including in-hospital mortality and inevitable death upon discharge). Subgroup analyses were conducted for the patients with follow-up visits. DNS diagnosis was based on outpatient department (OPD) records. Delayed neuropsychiatric syndrome (DNS) was defined as the development of new neuropsychiatric symptoms—such as cognitive impairment, mood disturbances, gait abnormalities, or urinary incontinence—occurring after a lucid interval of 2 to 40 days following acute carbon monoxide poisoning, in the absence of other identifiable causes. Diagnosis was based on clinical evaluation and supported by neuroimaging findings when available. The following demographic data were extracted from the CGMH electronic medical records: age, sex, intoxication mechanism, and vital signs (body temperature, heart rate, respiratory rate, blood pressure), O_2_ saturation laboratory examinations, and length of hospital stay.

### Data analysis

Continuous variables, including age, vital signs, laboratory examination results, GCS scores, and length of hospital stay, were presented as medians with interquartile ranges (Q1–Q3). Categorical data were presented as frequencies and percentages. The Mann–Whitney U test was used for continuous variable analysis, while the chi-square and Fisher’s exact tests were used for categorical data analysis. Receiver operating characteristic (ROC) curve analysis was used to determine the optimal cut-off values for various laboratory examinations, including WBC count, COHb and AST levels, and RDW. Among these, AST showed the highest area under the ROC curve (AUC). The optimal cutoff values for AST, along with measures of sensitivity, specificity, positive predictive value, and negative predictive value were analyzed. A two-tailed p-value <0.05 was considered statistically significant. All analyses were performed using SPSS for Windows (version 22.0; IBM Corp., Armonk, NY, USA; released in 2013).

### Language editing

The manuscript was initially written using a mix of Chinese and English. OpenAI's ChatGPT-4o was used for translations, and the resulting text was subsequently reviewed and edited by the authors to ensure content accuracy and integrity.

## Results

**Figure 1:**
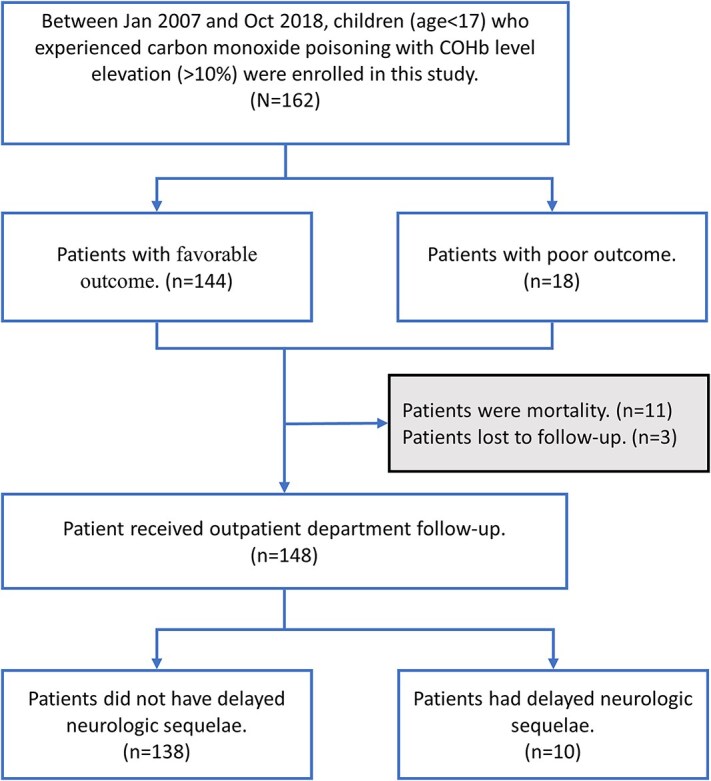
Flowchart of carbon monoxide patients’ enrollment and subgroup analysis. Poor outcome was defined as in-hospital mortality, impending death discharge, or GCS <13 at discharge.

Between January 2007 and October 2018, 162 patients aged <17 yr with COP and COHb levels greater than 10% were enrolled in this study (Fig. 1). Poor outcomes were defined as a GCS score < 13 at discharge or death. Eighteen patients (11.1%) met the definition of poor outcome; among them, 11 died (the additional data of 7 survivals are in the Supplementary Material 1). A total of 148 patients (91%) were discharged and followed-up in the OPD. Ten children were diagnosed with DNS during the follow-up.

The clinical characteristics of the patients are shown in Table 1. The primary sources of poisoning were incomplete fuel combustion (50.0%), fire scenes (8.0%), defective heaters (12.3%), and others (including diving and unknown reasons). Most patients were brought to the ED within the first hour of exposure (34.6%) or 1–6 h after exposure (55.6%). Lower oxygen saturation and lower GCS score were observed in the poor than in the favorable outcome group (SpO_2_: 95%–98% vs. 45%–97%, *P* = 0.03; and GCS: 15 vs. 3–9, *P* < 0.001 in the favorable vs. poor outcome group, respectively). Patients in the poor outcome group had longer length of in-hospital stay than those in the favorable outcome group (0.6 vs. 4.6 days, respectively, *P* < 0.001). Significant differences were found in WBC counts (11,800 vs. 19,300, *P* < 0.001), AST levels (28 vs. 56.5, *P* < 0.001), lactate levels (12.8 vs. 69.1, *P* = 0.039), RDW (9.7 vs. 86, *P* < 0.001), and troponin I levels (0 vs. 0.2, *P* = 0.003). No significant differences in COHb (p = 0.069) or Hb (p = 0.786) levels were observed between the two groups.

**Table 1:** Clinical characteristics of patients with carbon monoxide poisoning. (N = 162).

	Favorable outcome (n = 144)	Poor outcome (n = 18)	P-value
Age	9.5 (5–13)	8 (6–11)	0.659
Male sex	63 (43.8%)	8 (44.4%)	0.955
Intentional	7 (4.9%)	0 (0%)	0.431
Mechanism			
Incomplete combustion of fuels	76 (52.8%)	5 (27.8%)	<0.001
Fire scene	12 (8.3%)	1 (5.6%)	
Defective heaters	8 (5.6%)	12 (66.7%)	
Others	48 (33.3%)	0 (0%)	
From COP to emergency department			
< 1 h	53 (36.8%)	3 (16.7%)	0.252
1–6 h	78 (54.2%)	12 (66.7%)	
6–24 h	11 (7.6%)	3 (16.7%)	
> 72 h	2 (1.4%)	0 (0%)	
Vital signs during triage			
Body temperature (°C)	36.6 (36–37.1)	35 (32–36.4)	<0.001
Heart rate (beats/minute)	122 (106–134.5)	105.5 (0–135)	0.026
Respiratory rate (times/minute)	21 (20–24)	18 (0–24)	0.019
SpO2 (%)	97 (95–98)	95 (45–97)	0.030
MAP (mmHg)	84.5 (75–93.7)	60.7 (0–100.3)	0.037
GCS scores	15 (15–15)	3 (3–9)	<0.001
Admission	36 (25%)	15 (83.3%)	<0.001
Length of stay (days)	0.6 (0.4–1.3)	4.6 (1–10.1)	<0.001
Laboratory exam			
Carboxyhemoglobin (%)	21.4 (15.6–26.7)	29.7 (19.4–40.7)	0.069
White blood count (1,000/liter)	11.8 (8.6–14.6)	19.3 (12.2–31.2)	<0.001
Hemoglobin (grams/deciliter)	13.4 (12.3–14.1)	13.4 (12.1–14.9)	0.786
Platelets (1,000/microliter)	302.5 (255–364)	351.5 (285–417)	0.055
Power of hydrogen, pH	7.4 (7.4–7.4)	7 (6.8–7.4)	<0.001
Bicarbonate (mEq/liter)	22.5 (19.8–24.1)	17.4 (12.7–23.6)	0.024
BUN (milligrams/deciliter)	13 (10.1–14.4)	14.7 (11–19.5)	0.202
Creatinine (milligrams/deciliter)	0.5 (0.4–0.6)	0.9 (0.7–1.2)	<0.001
C-reactive protein (mg/liter)	0.5 (0.5–1.6)	0.3 (0.2–10.6)	0.450
Sodium (mEq/liter)	139 (138–140)	141 (139.2–143.1)	0.001
Potassium (mEq/liter)	3.7 (3.5–4)	4.1 (3.8–6.1)	0.003
AST (unit/liter)	28 (22–34)	56.5 (41.5–169.5)	<0.001
ALT (unit/liter)	16 (12–19)	97 (21–152)	0.001
Lactate (milligrams/deciliter)	12.8 (8.1–28.2)	69.1 (50.8–91.6)	0.039
Glucose (milligrams/deciliter)	113 (101–128.5)	301 (113–390)	0.019
RDW (femtoliters)	9.7 (0.7–31)	86 (41.5–274.2)	<0.001
CPK (unit/liter)	3.9 (0.5–24)	30 (11.2–91.6)	0.005
Troponin I (nanograms/milliliter)	0 (0–0)	0.2 (0–2.8)	0.003
Hyperbaric oxygen therapy	63 (43.8%)	2 (11.1%)	0.008
Experienced intubation	4 (2.8%)	15 (83.3%)	<0.001

Data are presented as number (percentage), mean ± standard deviation, or median (Q1-Q3).

Poor outcome was defined as GCS < 13 or mortality.

Abbreviations: COP, carbon monoxide poisoning; MAP, mean arterial pressure; SpO2, peripheral oxygen saturation; GCS, Glasgow coma scale; mEq, Milliequivalents; BUN, Blood urea nitrogen; AST, Aspartate aminotransferase; ALT, Alanine transaminase; RDW, Red cell distribution width; CPK, Creatine-phospho-kinase; OPD, out-patient department.

**Figure 2:**
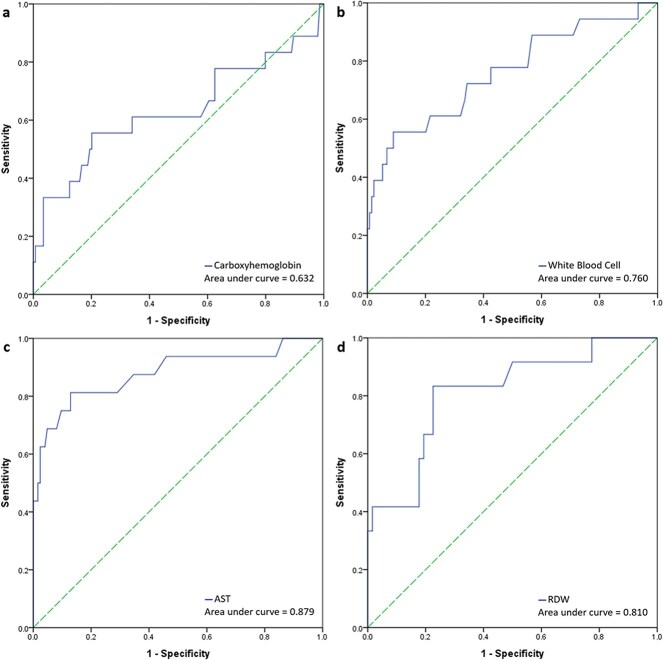
The receiver operating characteristic (ROC) curve of the a. Carboxyhemoglobin, b. white blood cell, c. aspartate aminotransferase (AST), and d. red cell distribution width (RDW).

Figure 2 shows the ROC curves of the four blood examination parameters. The AUC for COHb level, WBC count, AST level, and RDW were 0.595, 0.739, 0.859, and 0.755, respectively. AST level was further analyzed as a predictor of COP prognosis, as its AUC value was the highest among the AUC values of the other parameters (Table 2). The AST results were lacking for 22 children; therefore, the AST levels of only 140 patients were analyzed. An AST cutoff value of 38.5 could be used to predict poor outcomes in pediatric patients with COP (accuracy = 86.43%; 95% confidence interval [CI]: 79.62%–91.63%).

**Table 2:** The sensitivity and specificity of using AST level to estimate COP risk. (N = 140).

	Favorable outcome (n = 124)	Poor outcome (n = 16)
Low risk (AST < 38.5)	108	3
High risk (AST ≥ 38.5)	16	13
	Value	95% CI
Sensitivity	81.25%	54.35% to 95.95%
Specificity	87.10%	79.89% to 92.44%
Positive Predictive Value	44.83%	32.70% to 57.61%
Negative Predictive Value	97.30%	92.83% to 99.01%
Accuracy	86.43%	79.62% to 91.63%

A total 22 children did not exam AST. Abbreviations: AST, Aspartate aminotransferase; COP, Carbon monoxide poisoning.

A subgroup analysis was conducted in patients who underwent OPD follow-up after discharge. Of 148 patients who underwent follow-up visits, 10 were diagnosed with DNS (Table 3). Higher COHb levels and lower GCS scores were observed at discharge in patients with DNS than in those without DNS (COHb, *P* = 0.032; GCS score, *P* = 0.002). Additionally, blood urea nitrogen (BUN) and sodium levels were higher in patients with than in those without DNS (BUN, 14.8, *P* = 0.041; sodium, 142, *P* = 0.029). No significant differences were noted in WBC counts, AST levels, and RDW between the two groups. In a supplementary multivariate logistic regression including 62 patients with complete data, AST (1.052, 95% CI: 0.975–1.136), COHb (1.076, 95% CI: 0.997–1.162), and WBC (1.083, 95% CI: 0.910–1.288) showed a trend toward association with poor outcomes, though none reached statistical significance.

**Table 3:** Subgroup analysis of patients with outpatient department follow-up (N = 148).

	No delayed neurologic sequelae (n = 138)	Delayed neurologic sequelae (n = 10)	P-value
Age	9 (5–13)	11 (7–14)	0.358
Male sex	59 (42.8%)	4 (40%)	1.000
Hyperbaric oxygen therapy	58 (42%)	7 (70%)	0.106
Experienced intubation	5 (3.6%)	1 (10%)	0.348
Hospital length of stay (days)	0.6 (0.4–1.4)	1 (0.3–2.6)	0.285
GCS scores at discharge	15 (15–15)	15 (11–15)	0.002
Laboratory exam			
Carboxyhemoglobin (%)	21.1 (15.4–25.6)	31.5 (20.4–34.5)	0.032
White blood count (1,000/liter)	11.8 (8.7–14.7)	12 (7.3–14.9)	0.977
Power of hydrogen, pH	7.4 (7.4–7.4)	7.4 (7.3–7.4)	0.046
BUN (milligrams/deciliter)	12 (10–14)	14.8 (14.8–21.8)	0.041
Sodium (mEq/liter)	139 (138–140)	142 (140.2–144.5)	0.029
AST (unit/liter)	28 (22–34)	28 (24–36)	0.606
RDW (femtoliters)	12 (0.7–38)	27.1 (0.2–54)	0.676

Data are presented as number (percentage) or median (Q1-Q3).

Abbreviations: GCS, Glasgow coma scale; BUN, Blood urea nitrogen; AST, Aspartate aminotransferase.

## Discussion

This study enrolled 162 patients under the age of 17 yr who were admitted to the ED because of COP. The time from poisoning to ED arrival and the clinical and laboratory characteristics upon arrival were analyzed to elucidate the relationships between these parameters and patient outcomes. The primary causes of COP were incomplete fuel combustion and defective heaters, which aligned with the findings reported in previous studies on COP cases in Taiwan.^8^^,^^15^

CO exerts it adverse effect mainly on the nervous and cardiovascular systems.^7^^,^^8^^,^^23^ In our study, the group with poor outcomes presented with worse initial vital signs, such as lower SpO_2_, mean arterial pressure, and GCS scores compared to the group with favorable outcomes. These findings are consistent with previous studies.^18^^,^^19^ Regarding the length of hospital stay, patients in the poor outcome group had significantly longer hospital stays than those in the favorable outcome group. Additionally, a high proportion of patients in this group progressed to respiratory failure and required intubation. These results are consistent with those of Chang et al.^8^

Previous studies had revealed that higher COHb level was associated with more severe symptoms such as altered consciousness, seizures, and respiratory failure in patients with acute COP.^18^^,^^19^^,^^23^ However, COHb level was not a reliable predictor of prognosis in patients with COP. Chang et al.^8^ found that COHb levels could not be used to predict the occurrence of delayed or permanent neurological sequelae in patients aged <18 yr. Akcan et al.^18^ reported that COHb levels cannot predict a severe clinical course. Similar findings have been reported in adult patients with COP.^15^^,^^17^ Our study found that COHb level did not have a good ROC analysis result, which is compatible with the findings of previous studies.^15^ Recent studies, such as that by Kim et al. (2022), have developed and validated clinical nomograms incorporating multiple variables to predict delayed neuropsychiatric sequelae, emphasizing that COHb alone is insufficient for outcome prediction and should be interpreted alongside other clinical and biochemical markers.^21^ There are challenges in including COHb as a predictor for carbon monoxide poisoning, inducing poor outcomes.^24^

Akcan et al.^18^ and Guven et al.^19^ reported that WBC counts were significantly higher in pediatric patients with severe COP compared to those with favorable outcomes. Guven et al.^19^ also showed that higher RDW values can predict severe COP. In our study, WBC counts and RDW were significantly higher in the poor than in the favorable outcome group, and moderate values for poor outcome prediction were found via ROC analysis, with AUCs of 0.760 for WBC counts and 0.810 for RDW.

Previous studies found that elevated lactate and troponin levels were associated with a greater severity of COP and might result primarily from tissue hypoxia and myocardial injury.^18^^,^^25–27^ İpek et al.^22^ explored that elevation of NT-proBNP levels might be detected earlier than that of troponin I levels in patients with COP. The accuracy of the COP diagnosis can be improved when evaluated using a combination of troponin and BNP levels.^22^^,^^28^ The liver receives approximately 20%–25% of the cardiac output, with 70% from the portal vein and 30% via the hepatic artery. Owing to its dual blood supply, the liver is generally protected from ischemic injury.^29^ Anoxic hepatic injury with a significant elevation of AST levels in an adult with severe COP was reported by Watson et al.^30^ Elevated AST levels in pediatric carbon monoxide poisoning (COP) likely reflect systemic hypoxic injury rather than isolated liver damage. AST is present in the liver, heart, and muscle, and rises in response to cellular damage from hypoxia, inflammation, and oxidative stress, hallmarks of CO toxicity.^2^ The liver’s susceptibility to hypoxic injury can contribute to AST elevation, but additional sources such as myocardial or skeletal muscle injury may also play a role.^29^^,^^31^ Kim et al. reported AST elevation in severe COP cases, supporting its utility as a surrogate marker of global injury severity.^32^ Thus, elevated AST may serve as a surrogate marker of global hypoxic injury, aligning with the pathophysiology of CO poisoning. Its prognostic relevance may help identify pediatric patients at higher risk of adverse outcomes. Subclinical hepatic injury, defined as a serum alanine aminotransferase (ALT) level above the upper limit of normal (ULN) but less than three times the ULN, was reported to be 14.3% in adult patients. In addition, CO-induced hepatic injury, defined as an ALT level greater than three times the ULN, was reported to be 1.6% in a study conducted by Kim et al.^32^; however, the association between hepatic injury and mortality was not significant. A higher incidence of intensive care unit admission and other complications, such as acute kidney injury and cardiomyopathy, was observed in the hepatic injury group than in the no hepatic injury group. Studies on the prevalence of acute liver dysfunction in pediatric patients with COP are lacking. Our study discovered that serum AST level was a better predictor of poor outcomes in pediatric patients with COP than WBC count and RDW (AUC = 0.879). AST is less specific to liver injury than ALT and increased level may be found in myocardial infarction, rhabdomyolysis or tissue ischemia.^31^^,^^33^ To the best of our knowledge, this is the first study to reveal the relationship between serum AST levels and adverse outcomes in pediatric patients with COP. Hafez et al. suggested that the serum S-100β level at admission may serve as an indicator of poisoning severity and a potential predictor for the development of DNS following carbon monoxide exposure, thus aiding in decisions regarding HBO therapy.^34^ However, our study did not include measurements of serum S-100β levels. Elmansy et al. reported that an early glucose-to-potassium (Glu/K) ratio could serve as an effective, reliable, and convenient biomarker for predicting mortality, delayed neuropsychiatric sequelae (DNS), and the need for mechanical ventilation in patients with acute carbon monoxide poisoning.^35^ In our study, the Glu/K ratio also demonstrated fair predictive ability, with an AUROC reaching 0.717.

DNS are potential complications of acute COP. DNS typically occurs within days to weeks of COP and presents with symptoms such as cognitive impairment, akinetic mutism, gait ataxia, and Parkinsonism-like movement disorders.^5^^,^^36^ Previous studies have shown that low GCS scores and intubation are risk factors for the development of DNS.^8^^,^^15^ The COHb levels were not strongly associated with the occurrence of DNS. The effectiveness of HBOT in preventing DNS remains controversial.^8^^,^^13^^,^^15^^,^^23^^,^^37^ In the present study, higher COHb levels at presentation and lower GCS scores at discharge were associated with the occurrence of DNS. Intubation and HBOT were not strongly related to the occurrence of DNS. The small number of intubated pediatric patients with COP, exclusion of HBOT in intubated patients in the CGMH hospital system due to the equipment setting, and exclusion of patients who died of acute COP might have confounded the results. The lack of significant AST elevation between DNS and non-DNS groups may be due to the exclusion of patients who died during hospitalization, as they had markedly elevated AST levels. Since our DNS analysis was based on outpatient follow-up, deceased patients could not be included, potentially attenuating the observed difference in AST levels.

### Limitations

This study has a few limitations. First, this was a retrospective study. Although this study reflected a multicenter experience, the data were collected entirely in Taiwan and within the branches of the CGMH system, which might have resulted in high homogeneity. Second, patients with suspected CO exposure and a COHb level < 10% were excluded. Third, the results may overestimate the predictive value of AST, especially given the retrospective nature of the study.

## Conclusion

To summarize, we found that elevated AST levels in pediatric patients with COP had a higher AUC (0.879) than other blood biomarkers as reported in previous studies, suggesting that AST is a more reliable predictor of poor outcomes. Using an AST cut-off value of 38.5 U/L, the sensitivity (81.25%, 95% CI: 54.35%–95.95%) and specificity (87.10%, 95% CI: 79.89%–92.44%) were favorable predictors of poor outcomes. Although AST was not significant in predicting the occurrence of DNS specifically, it remains a useful early marker for identifying patients at risk of overall poor prognosis. These findings suggest that AST could be incorporated into early triage and risk stratification in pediatric COP, and future prospective studies are warranted to validate its utility and explore its integration with other biomarkers.

## Supplementary Material

the_patients_prgonosis_revised_tfaf154

## Data Availability

The datasets collected in current study are not publicly available due to patient privacy but are available from the corresponding author upon reasonable request.
